# Spatio-Temporal Pattern and Socio-Economic Factors of Bacillary Dysentery at County Level in Sichuan Province, China

**DOI:** 10.1038/srep15264

**Published:** 2015-10-15

**Authors:** Yue Ma, Tao Zhang, Lei Liu, Qiang Lv, Fei Yin

**Affiliations:** 1 West China School of Public Health, Sichuan University, Chengdu, Sichuan, People’s Republic of China; 2 Sichuan Center for Disease Control and Prevention, Chengdu, Sichuan, People’s Republic of China

## Abstract

Bacillary dysentery (BD) remains a big public health problem in China. Effective spatio-temporal monitoring of BD incidence is important for successful implementation of control and prevention measures. This study aimed to examine the spatio-temporal pattern of BD and analyze socio-economic factors that may affect BD incidence in Sichuan province, China. Firstly, we used space-time scan statistic to detect the high risk spatio-temporal clusters in each year. Then, bivariate spatial correlation and Bayesian spatio-temporal model were utilized to examine the associations between the socio-economic factors and BD incidence. Spatio-temporal clusters of BD were mainly located in the northern-southern belt of the midwest area of Sichuan province. The proportion of primary industry, the proportion of rural population and the rates of BD incidence show statistically significant positive correlation. The proportion of secondary industry, proportion of tertiary Industry, number of beds in hospitals per thousand persons, medical and technical personnel per thousand persons, per capital GDP and the rate of BD incidence show statistically significant negative correlation. The best fitting spatio-temporal model showed that medical and technical personnel per thousand persons and per capital GDP were significantly negative related to the risk of BD.

Bacillary dysentery (BD), caused by different species of Shigella bacteria, is a bacterial infection of intestines which may result in severe diarrhea. The major symptoms of BD are fever, abdominal pain, and uncontrolled loose or watery stools containing visible red blood^1^. The infection can be transmitted by the fecal-oral route via contaminated water, food or person-to-person contact. BD is a major public health issue in many developing countries in the world. And it remains a major public health concern in China^2^. Although the morbidity and mortality of BD in China have been declining progressively in the past decade, a considerable disease burden still exists^3^,^4^. According to the national surveillance system of China, there were 205,972 bacillary dysentery cases reported in 2012, with an incidence rate of 15.29 per 100,000^5^. Sichuan province, located in south-west China, is the second largest province in China. Sichuan province had a relatively high incidence of BD, which is one of the most frequently reported infectious disease in Sichuan province in recent years^6^,^7^.

The results of many studies have indicated that BD are not equally distributed across geographic areas^8^,^9^,^10^. In addition, seasonality in the incidence of BD has been reported in previous studies^11^,^12^,^13^. A better understanding of the spatio-temporal patterns of BD would help to identify high risk areas and periods, and thus help health officials in designing more location-specific public health interventions to control or prevent BD. However, spatial and temporal models are insufficient for exploring transmission models. Risk factors can also be used to determine how interventional work should be conducted. The incidence of BD is related to changes in biology, socio-economic status and environment over space and time^14^. The relationship between meteorological factors and BD has been reported in many studies^11^,^12^,^13^,^15^. But the association between socio-economic variables and BD is still far from clear, with very few studies examining the quantitative relationship between socio-economic factors and BD^16^.

This is the first research targeted at the spatio-temporal characteristics of BD in Sichuan province over the last eleven years (2004 to 2014). The authors aimed to explore the spatio-temporal pattern of BD and socio-economic factors that affect the incidence of BD. The spatial, temporal and spatio-temporal analyses were conducted to determine high risk regions of BD, thus to provide information on appropriate allocation of public health resources for better disease control and prevention.

## Results

Between January 2004 and December 2014, a total of 201,149 BD cases were reported in Sichuan province. The annual incidence ranged from 9.16 per 100,000 to 38.80 per 100,000 population, with an average annual incidence rate of 22.12 per 100,000 population. Table 1 showed the incidence rate of BD by year. The incidence rate increased from 29.44 per 100,000 in 2004 to 38.80 per 100,000 in 2006, and then declined during 2007–2014. In 2014, the incidence rate was 9.16 cases per 100,000 population, less than one third of what it was in 2004. Of 201,149 BD cases, 109,071 (54.22%) were males and 92,078 (45.78%) were females, with a male-to-female sex ratio 1.18.

**Figure 1:**
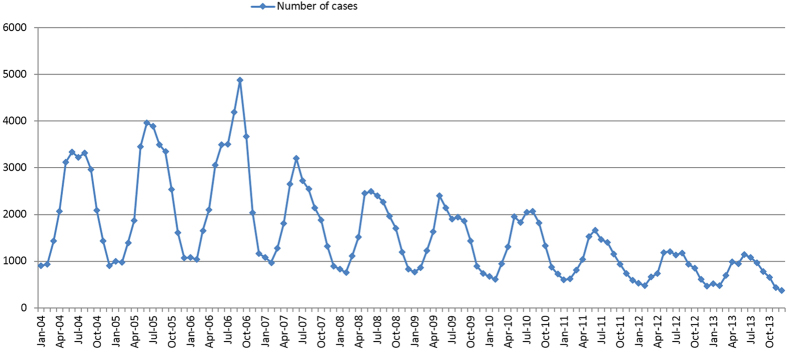
Monthly distributions of reported BD cases in Sichuan, China, from Jan, 2004 to Dec, 2014.

**Figure 2:**
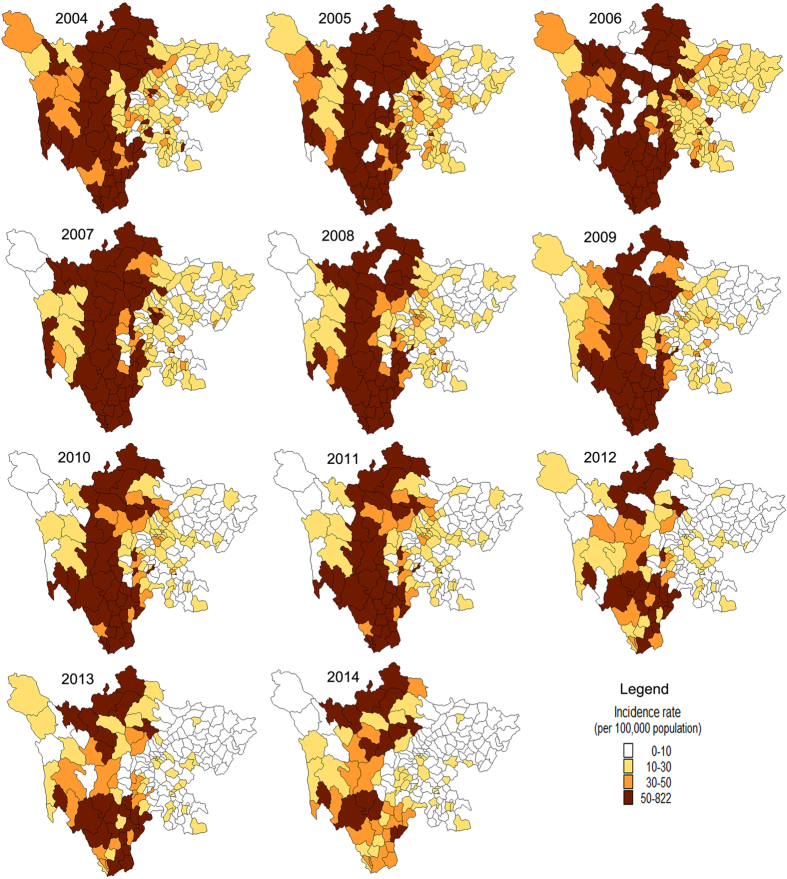
Yearly incidence rates (per 100,000) of BD at the county level in Sichuan, China, 2004–2014 (created with MapInfo Professional software version 7.0).

Figure 1 illustrated the monthly distribution of BD cases. Clear seasonality peaks were observed. The seasonal peak occurred between May and September for each year. The spatial distribution of annual incidence rate of BD cases is shown in Fig. 2. It clearly indicated that the spatial distribution of BD was heterogeneous at county level. Relatively high incidence rates appeared in the northern-southern belt of the midwest region.

**Figure 3:**
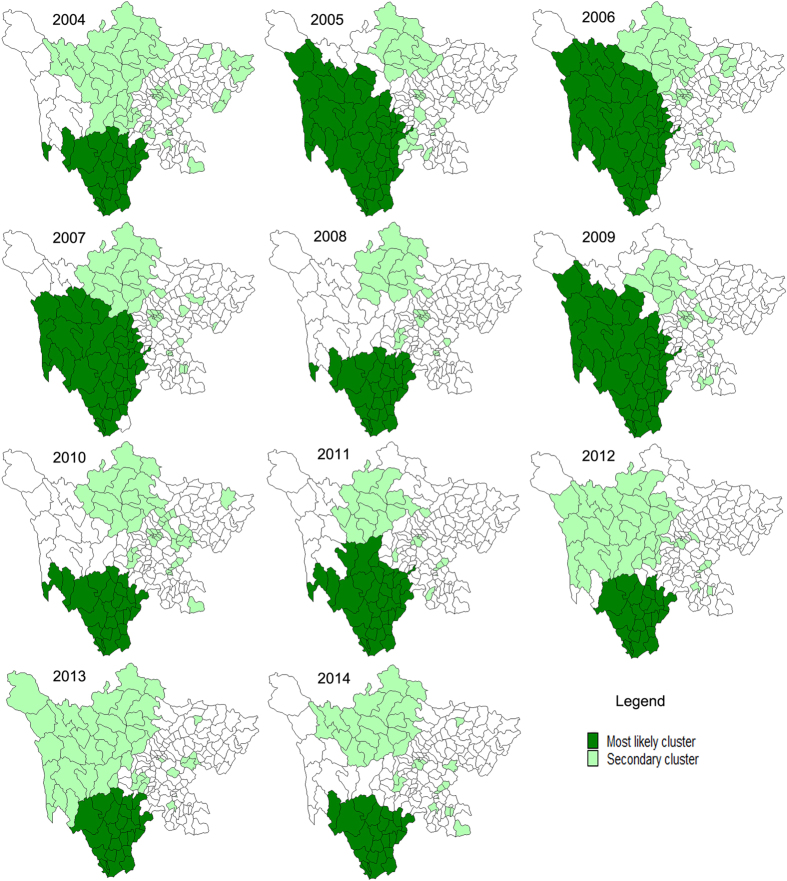
Spatial-temporal clusters of BD in Sichuan, China, 2004–2014 (created with MapInfo Professional software version 7.0).

Spatial-temporal cluster analysis identified one most likely cluster and fifteen secondary clusters in 2004. The most likely cluster included 27 counties, of which the cluster center was (26.40N, 101.64E) and the cluster radius was 328.39km. The cluster time was April to Setemper in 2004, and the average annual incidence rate inside the window was 175.6 per 100,000 with the relative risk (RR) value 7.29 (P < 0.001). Similarly, we could detect most likely clusters in other ten years (Fig. 3 and Table 2). The cluster centers were located in Panzhihua prefecture in 2004, 2008, 2012 and 2014. The cluster centers were located in Ganzhi prefecture in 2005–2007 and 2009. The cluster centers were located in Liangshan prefecture in 2010, 2011, and 2013. Nineteen counties were always included in the most likely cluters during 2004–2014, most of which were located in Liangshan prefecture (68.42%). Some of these counties deserve more attention. For instance, Muli county. Muli is in Liangshan prefecture which is located in southern Sichuan Province. Muli was included in the most likely cluster each year from 2004–2014. The incidence of BD in Muli increased from 76.16 to 103.85 cases per 100,000 people between 2007 and 2014, while the incidence rate of most counties in Sichuan province decreased a lot during this period. In each year, apart from the most likely cluster, several secondary clusters were also detected (Fig. 3). Most of clusters were located in the northern-southern belt of the midwest area of Sichuan province.

Table 3 showed the spatial correlation between the socio-economic factors and the incidence rate of BD at county level in Sichuan province in 2012. The proportion of primary industry and the proportion of rural population was positive correlated with the incidence rate of BD. The proportion of secondary industry, the proportion of tertiary industry and the incidence rate of BD showed a negative correlation. Possible reasons were that in Sichuan province the agricultural machinery production level was not high, and the low-level sanitary conditions of farmers’ living and working environment caused susceptibility to BD. While workers of secondary industry and tertiary industry have relative better sanitary conditions and therefore are less susceptible to BD. Number of beds in hospitals per thousand persons, medical and technical personnel per thousand persons were positively correlated with BD incidence. The two factors are representative of the level of medical conditions. This result suggests that better medical conditions might helpful for the reducing of disease transmission. Per capital GDP and the incidence rates of BD showed a negative correlation. Per capita GDP is a proxy variable for the level of economic development. The results indicated that good economy was helpful for the improvement of public health condition and reduction BD transmission.

**Figure 4:**
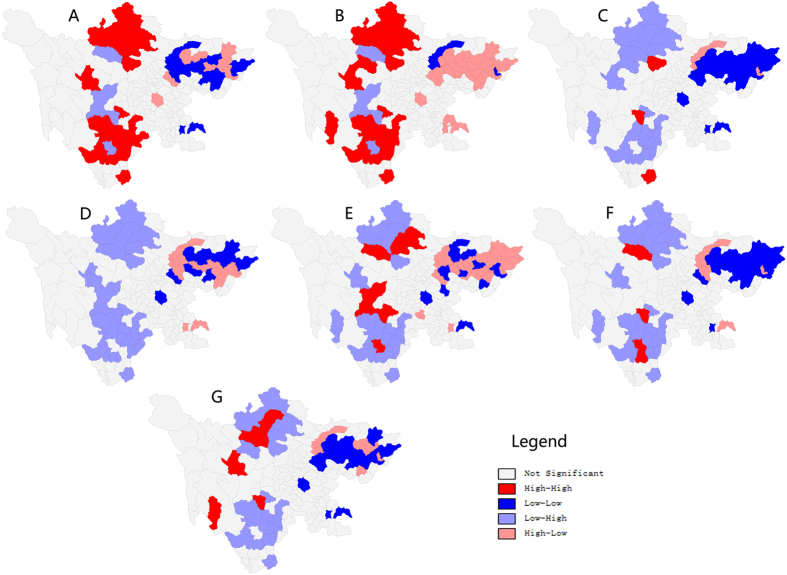
Bivariate LISA cluster maps of socio-economic factors and BD incidence at county level in Sichuan province, 2012. (**A**) Bivariate LISA cluster map of the proportion of primary industry and BD incidence. (**B**) Bivariate LISA cluster map of the proportion of rural population and BD incidence. (**C**) Bivariate LISA cluster map of Per capita GDP and BD incidence. (**D**) Bivariate LISA cluster map of the proportion of secondary industry and BD incidence. (**E**) Bivariate LISA cluster map of the proportion of tertiary industry and BD incidence. (**F**) Bivariate LISA cluster map of number of beds in hospitals per thousand persons and BD incidence. (**G**) Bivariate LISA cluster map of medical and technical personnel per thousand persons and BD incidence (created with GeoDa software version 1.3.28).

In Fig. 4A, the “high-high” and “low-low” clusters indicated a significant positive spatial correlation between the proportion of primary industry and the incidence rates of BD. The results provided evidence that the regions that had higher proportion of primary industry were also more likely to have higher BD incidence rates (“high-high”). Similarly, we observed some regions where both proportion of primary industry as well as BD incidence rates were low (“low-low”). A similar pattern was observed in Fig. 4B. The geographic regions that were marked by high rural population proportion had high levels of BD incidence rates. Figure 4C provided evidence that the regions that were poor in economic resources were also more likely to have higher BD incidence rates (“high-low”). Similarly, there were some regions which have better economic resources and lower BD incidence rates (“low-high”). Similar patterns were observed in Fig. 4D–G. The geographic regions that had lower proportion of secondary industry and tertiary industry and were poor in medical resources had high levels of BD incidence rates.

In Table 4, the parameter estimates for association and the corresponding credible interval of Bayesian spatio-temporal model were presented. The best model included two social-economic variables: medical and technical personnel per thousand persons and per capital GDP. The results indicated that medical and technical personnel per thousand persons and per capital GDP were significantly related to the risk of BD, as their 95% credible intervals were less than zero. The model with social-economic variables had a greater fit (DIC = 42202.64) than the model without social-economic variables (DIC = 40931.59). GDP per capita and technical personnel per thousand persons were negatively associated with the disease incidence. An increase of 1000 yuan in the per capita GDP was associated with a decrease of around 2% in the relative risk of BD. An increase of one person in the medical and technical personnel per thousand persons was associated with a decrease of around 1% in the relative risk of BD.

In our study, all BD cases were clinical or laboratory-confirmed and reported by hospital diagnostic. Among the total BD cases, 59734 (29.7%) were laboratory-confirmed cases. We have run the same spatial-temporal analyses on laboratory-confirmed cases of BD. Results of space-time cluster dection were showed in Supplementary Fig S1 and Supplementary Table S1 online. Supplementary Table S2 and Supplementary Fig S2 showed the spatial correlation between the socio-economic factors and the incidence rate of BD at county level in Sichuan province in 2012. In Supplementary Table S3 online, the parameter estimates for association and the corresponding credible interval of Bayesian spatio-temporal were presented.

## Discussion

In our study, the temporal analysis showed that most cases occurred in summer and autumn. This was consistent with the findings in other areas of China. Similar seasonal variations were also found in Changsha city and Jiangsu Province^8^,^11^. However, there was a little difference. For example, the seasonal peak was observed from June to October in Changsha city, which is one month later than that in Sichuan province. Epidemiologists have long been perplexed by the causes of seasonality in infectious diseases of humans. Possibly there is no single theory which could explain this phenomenon^17^,^18^. Environment changes, especially climate changes, have been mostly implicated. Several previous studies demonstrated that temperature played an important role in the seasonality of BD^11^,^12^,^13^,^19^,^20^,^21^. During the high epidemic months (May-September), temperature was higher than that in other months. Rising temperature could increase the incubation and survival of Shigella in the environment. The optimum temperature for the growth of Shigella is 37 °C^22^. In addition, the housefly population density also increases during warm days^23^.

During the eleven-year study period, BD incidence in Sichuan province markedly increased during 2004–2006, and then decreased during 2007–2014. The spatio-temporal scanning results showed that the centers of the most likely cluster were all located in the southwest Sichuan province during the whole study period. The most likely cluster covered counties located in Panzhihua prefecture, Ganzhi prefecture, Liangshan prefecture and its neighboring areas for each year. The most likely cluster detected in 2012 included 23 counties that were mostly located in Liangshan prefecture. Liangshan prefecture, officially the Liangshan Yi autonomous prefecture, has the largest population of ethnic Yi nationally. It is the less developed area in Sichuan province. The result was consistent with the finding of the spatial correlation in which economic development played very important roles in the incidence of BD.

In this study, we used bivariate spatial correlation to examine the spatial relationship between the socio-economic factors and the BD incidence for the geographic regions of Sichuan province. The result indicated that proportion of primary industry and proportion of rural population had a positive association with the BD incidence. And the other five socio-economic factors were positively associated with the BD incidence. Furthermore, bivariate Local Moran’s I revealed the local variations of the spatial dependence between the socio-economic factors and the BD incidence across Sichuan province.

The results of Bayesian spatio-temporal model showed that medical and technical personnel per thousand persons and per capital GDP were significantly negatively related to the risk of BD. Progress in economy might helpful for the improvement of hygiene and better access to sanitary water and food. This was consistent with the previous findings. Ferrer *et al.* reported economic factors as one of the factors determining diarrhea occurrence^24^. Tang *et al.* reported that people with higher family income had a lower BD incidence rate^8^. In addition, better medical conditions might helpful for the reducing of disease transmission. Preventative strategies should be concentrated in areas with poor economic and medical conditions.

A few limitations are deserved to mention: 1) The underreporting of BD cases is a potential limitation of our study. Some BD cases do not seek health care because they are asymptomatic or their symptoms are mild. The reporting homogeneous among the various counties of Sichuan province is an important parameter when drawing comparisons on the burden of BD in the various counties. However, this parameter haven’t been taken into account in this study because the under-reporting rate of various counties in Sichuan province is not available. 2) In our study, we used county as the geographic unit of spatial analysis. However, finer geographic units may provide more useful information which could help health officials to devise more comprehensive strategies. A smaller spatial unit scale (e.g. township level) could be utilized in further research. For instance, the analysis could focus on Muli county and its neighboring counties to detect spatio-temporal clusters with higher burdens of BD at township level. 3) We could not differentiate the pathogens of BD cases reported to the CISDCP system. Therefore, we were unable to examine the specific impacts of socio-economic indicators on different pathogens. 4) In this study, we only examined the relationship between the incidence of BD and socio-economic variables. In further research, more thorough study about the driving forces and risk factors (climate, geography and environment) that contribute to transmission of BD are needed.

In conclusion, our study provides a good understanding of the spatio-temporal distribution of BD in Sichuan province. Allocating more resources to high-risk locations at suitable times might help to reduce BD incidence more effectively. Our results provide evidence that socio-economic factors were spatial correlated with the incidence rate of BD. The success of BD intervention strategies could benefit from giving more consideration to local social and economic conditions.

## Materials and Methods

Sichuan province is located in south-west China between longitude 98.31E to 107.99E and latitude 26.40N to 33.68N. It is an inland province with a population of approximately 80 million people. Sichuan province covers an area of 485,000 km^2^, which is divided into 21 prefectures and 180 counties.

Records on BD cases in Sichuan Province from 2004 to 2014 were obtained from the China Information System for Disease Control and Prevention (CISDCP), which is a real-time web-based notifiable diseases reporting system. In our study, all BD cases were clinical or laboratory-confirmed and reported by hospital diagnostic. Demographic information was obtained from the National Bureau of Statistics of China. The socio-economic data from 2004 to 2012 were collected from Sichuan Statistical Yearbook. All collected data were geographically referenced based on 180 counties of Sichuan province, i.e., 180 spatial units for analysis.

A retrospective space-time scan statistic based on the discrete Poisson model was applied to detect high risk space-time clusters of BD cases within Sichuan province^25^. The spatio-temporal cluster analysis was conducted by using SaTScan software (version 9.2). The space-time scan statistic was defined by a cylindrical window with a circle indicating a geographic base and with height representing to time. The base of the cylinder indicated the underlying clustering areas, and the height represented the time period of the potential clusters. The cylindrical window was then moved over the study areas and periods to detect potential spatio-temporal clusters. For each scanning window, the difference of the incidence inside and outside the windows was calculated by log likelihood ratio (LLR). Monte Carlo testing was utilized to determine statistical significance of clusters. Scanning window with the maximum LLR was considered as the most likely cluster, indicating that it was least likely to have occurred by chance. In addition to the most likely cluster, other scan windows where the LLR values were statistically significant were identified as secondary clusters and ranked according to their likelihood ratio test statistic.

For this analysis, yearly scans were performed to control the time trend and to detect changes in spatio-temporal clustering during the whole study period. For each year, we used 180 counties of Sichuan province as spatial units, and 12 months from January to December as time units. To ensure sufficient statistical power, the number of Monte Carlo replications was set to 999. Statistical significance of the clusters was defined as a p-value less than 0.05.

Bivariate Moran’s I statistic was used to describe the spatial correlation between the social-economic factors and the incidence rate of BD in Sichuan province^26^. Firstly, a global bivariate spatial correlation analysis was applied in order to calculate a single measurement of spatial correlation between the incidence rate of BD and the social-economic variables. Then, a local bivariate spatial correlation analysis was adopted to identify local patterns of spatial associations which was based on the local indicator of spatial analysis – LISA approach^27^. The standardized first-order contiguity queen neighbors were used as the definition of neighbors in our study. Significance of the test statistic was assessed with a Monte Carlo P value generated using 999 random permutations. The bivariate spatial correlation analysis was conducted by using GeoDa software (version 1.3.28).

In our study, Bayesian spatio-temporal model was used to quantify the association between the incidence of BD and socio-economic variables from 2004 to 2012. The Bayesian spatio-temporal model can be expressed as^28^,^29^:


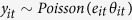



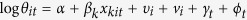


where *i* is an index for the spatial units (county), and *t* is an index for the time periods (year); 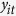 is the observed count; 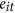 is the expected count of cases adjusted for age and gender. It is calculated by applying the provincial crude incidence rate to the county-specific age and gender distributions; 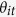 is the spatiotemporal-specific relative risk of disease; *α* is the intercept quantifying the average Poisson relative risk in the whole of Sichuan province; 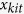 is the socio-economic variable; 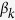 is the regression coefficient of socio-economic variable; 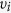 is the spatially structured random effect for county *i*, accounting for the assumption that geographically close areas are more related than distant areas; 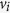 is the non-spatial random effect for county *i;* 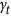 is the temporally structured effect for year *t*; 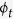 is the unstructured temporal effect for year *t.*

All random effects were modeled, and default minimally informative priors were specified^30^. Firstly, the spatial effect was modeled using an intrinsic conditional autoregressive structure. Secondly, the temporally structured effect was modeled dynamically through a time neighboring structure. Finally, the unstructured spatial and temporal effects were both specified by Gaussian models with a mean of zero. The Gamma (1, 0.0005) was chosen as the prior for the precision of the above Gaussian random effects.

Model parameters were estimated using integrated nested Laplace approximations (INLA), a method for approximate Bayesian inference in structured additive regression models with latent Gaussian models. INLA outperforms traditional Markov Chain Monte Carlo (MCMC) method in terms of computational time, while providing very accurate results^31^. The performance of models were compared using the deviance information (DIC). The model with the lowest DIC indicates the best trade-off between the model fit and complexity^32^. The INLA package in the R software (version 3.1.1) was used for the Bayesian spatio-temporal modeling.

## Additional Information

**How to cite this article**: Ma, Y. *et al.* Spatio-Temporal Pattern and Socio-Economic Factors of Bacillary Dysentery at County Level in Sichuan Province, China. *Sci. Rep.* **5**, 15264; doi: 10.1038/srep15264 (2015).

## Supplementary Material

Supplementary Information

## Figures and Tables

**Table 1:** Incidence of BD in Sichuan Province, 2004–2014.

Year	Number of cases	Incidence rate(/10^5^)
2004	25711	29.44
2005	28588	32.60
2006	31866	38.80
2007	22485	27.62
2008	19521	24.02
2009	17793	21.86
2010	16210	19.82
2011	12541	15.59
2012	9986	12.37
2013	9057	11.23
2014	7391	9.16
Total	201149	22.12

**Table 2:** The most likely clusters of BD in Sichuan Province, China, 2004–2014.

Scan timeframe	Cluster time	Cluster center/Radius	Annual cases/10^5^	*LLR*	*RR*	*P*
2004/1/1–2004/12/31	2004/4/1–2004/9/30	(26.40N, 101.64E)/328.39 km	175.6	5547.38	7.29	<0.001
2005/1/1–2005/12/31	2005/4/1–2005/9/31	(28.54N, 99.34E)/408.63 km	196.9	9136.10	8.09	<0.001
2006/1/1–2006/12/31	2006/5/1–2006/10/31	(29.11N, 99.72E)/369.42 km	174.0	5680.76	5.51	<0.001
2007/1/1–2007/12/31	2007/4/1–2007/9/30	(28.73N, 100.29E)/323.84 km	136.8	4872.49	6.23	<0.001
2008/1/1–2008/12/31	2008/4/1–2008/9/30	(26.40N, 101.64E)/329.31 km	177.9	6451.37	9.73	<0.001
2009/1/1–2009/12/31	2009/4/1–2009/9/30	(28.54N, 99.34E)/405.32 km	138.0	5880.38	8.53	<0.001
2010/1/1–2010/12/31	2010/3/1–2010/8/31	(27.69N, 101.39E)/226.44 km	164.6	6289.79	11.18	<0.001
2011/1/1–2011/12/31	2011/4/1–2011/9/30	(27.69N, 101.39E)/253.85 km	102.2	4096.35	8.77	<0.001
2012/1/1–2012/12/31	2012/5/1–2012/10/31	(26.53N, 101.85E)/285.81 km	86.0	2994.07	8.97	<0.001
2013/1/1–2013/12/31	2013/3/1–2013/8/31	(27.85N, 102.09E)/170.40 km	77.1	2791.44	8.94	<0.001
2014/1/1–2014/12/31	2014/4/1–2014/9/30	(26.40N, 101.64E)/290.91 km	58.0	1803.42	7.86	<0.001

**Table 3:** Spatial correlation between the socio-economic factors and the incidence of BD, 2012.

Index	Moran’I	P-value
Proportion of Primary Industry	0.3119	0.001
Proportion of Secondary Industry	−0.3464	0.001
Proportion of Tertiary Industry	−0.1674	0.001
Proportion of Rural Population	0.1370	0.001
Number of Beds in Hospitals per Thousand Persons	−0.0918	0.006
Medical and Technical Personnel per Thousand Persons	−0.0803	0.002
Per Capital GDP	−0.1177	0.001

**Table 4:** Parameter estimates of Bayesian spatio-temporal model with social-economic variables.

Variables	*β*	*e*^*β*^	SD	95% Credible interval	
				2.5%	97.5%
Intercept	0.2508	1.2851	0.0119	0.2274	0.2741
MTP	−0.0087	0.9913	0.0026	−0.0138	−0.0036
GDP	−0.0198	0.9804	0.0006	−0.0210	−0.0186

Note: MTP is medical and technical personnel per thousand persons; GDP is per capital GDP.
