# Supplementary material for: Spatio-Temporal Pattern and Socio-Economic Factors of Bacillary Dysentery at County Level in Sichuan Province, China
Source: Sci Rep. 2015 Oct 15;5:15264. doi: 10.1038/srep15264 (PMC4606827; doi:10.1038/srep15264)
Supplement: Supplementary Information [file srep15264-s1.doc]

**Title:**

Spatio-Temporal Pattern and Socio-Economic Factors of Bacillary Dysentery at County Level in Sichuan Province, China

**Authors:**

Yue MaA
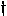
, Tao ZhangA
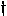
, Lei Liu^B^, Qiang Lv^B^ and Fei Yin^A^*

^A^ West China School of Public Health, Sichuan University, Chengdu, Sichuan, People’s Republic of China

^B^ Sichuan Center for Disease Control and Prevention, Chengdu, Sichuan, People’s Republic of China

*Corresponding author. Email: [westsilverhx@163.com](mailto:westsilverhx@163.com)


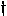
 The two authors, Yue Ma and Tao Zhang contributed equally to this work.

Supplementary Table S1. The most likely clusters of BD in Sichuan Province, China, 2004–2014.

| Scan timeframe | Cluster time | Cluster center/Radius | Annual  cases/10^5^ | *LLR* | *RR* | *P* |
| --- | --- | --- | --- | --- | --- | --- |
| 2004/1/1–2004/12/31 | 2004/3/1–2004/8/31 | (27.53N, 102.55E)/165.56 km | 48.3 | 1288.45 | 6.28 | <0.001 |
| 2005/1/1–2005/12/31 | 2005/5/1–2005/10/31 | (28.42N, 100.90E)/247.39 km | 51.1 | 1888.89 | 8.25 | <0.001 |
| 2006/1/1–2006/12/31 | 2006/5/1–2006/10/31 | (31.02N, 99.27E)/423.02 km | 52.8 | 1787.53 | 6.25 | <0.001 |
| 2007/1/1–2007/12/31 | 2007/5/1–2007/10/31 | (28.56N, 102.04E)/98.08 km | 52.4 | 641.91 | 9.06 | <0.001 |
| 2008/1/1–2008/12/31 | 2008/5/1–2008/10/31 | (28.56N, 102.04E)/98.08 km | 100.7 | 1759.78 | 17.16 | <0.001 |
| 2009/1/1–2009/12/31 | 2009/4/1–2009/9/30 | (28.56N, 102.04E)/98.08 km | 104.8 | 1885.82 | 18.26 | <0.001 |
| 2010/1/1–2010/12/31 | 2010/3/1–2010/8/31 | (27.60N, 102.89E)/154.51 km | 69.8 | 2531.48 | 13.70 | <0.001 |
| 2011/1/1–2011/12/31 | 2011/4/1–2011/9/30 | (27.67N, 103.23E)/160.89 km | 39.0 | 1369.14 | 9.80 | <0.001 |
| 2012/1/1–2012/12/31 | 2012/3/1–2012/8/31 | (27.60N, 102.89E)/136.65 km | 53.7 | 1842.73 | 15.87 | <0.001 |
| 2013/1/1–2013/12/31 | 2013/3/1–2013/8/31 | (27.60N, 102.89E)/136.65 km | 47.7 | 1615.96 | 15.41 | <0.001 |
| 2014/1/1–2014/12/31 | 2014/4/1–2014/9/30 | (27.60N, 102.89E)/136.65 km | 32.2 | 952.87 | 11.84 | <0.001 |

Supplementary Table S2. Spatial correlation between the socio-economic factors and the incidence of BD, 2012.

| Index | Moran’I | P-value |
| --- | --- | --- |
| Proportion of Primary Industry | 0.2842 | 0.001 |
| Proportion of Secondary Industry | -0.2529 | 0.001 |
| Proportion of Tertiary Industry | -0.2140 | 0.001 |
| Proportion of Rural Population | 0.1467 | 0.001 |
| Number of Beds in Hospitals per Thousand Persons | -0.1170 | 0.001 |
| Medical and Technical Personnel per Thousand Persons | -0.1309 | 0.001 |
| Per Capital GDP | -0.0822 | 0.008 |

Supplementary Table S3. Parameter estimates of Bayesian spatio-temporal model with social-economic variables

| Variables | $\beta$ | $e^{\beta}$ | SD | 95% Credible interval | |
| --- | --- | --- | --- | --- | --- |
|  |  |  |  | 2.5% | 97.5% |
| Intercept | 0.2002 | 1.2216 | 0.0473 | 0.1066 | 0.2942 |
| MTP | -0.0105 | 0.9896 | 0.0047 | -0.0198 | -0.0012 |
| GDP | -0.0311 | 0.9694 | 0.0012 | -0.0334 | -0.0288 |

Note: MTP is medical and technical personnel per thousand persons; GDP is per capital GDP.


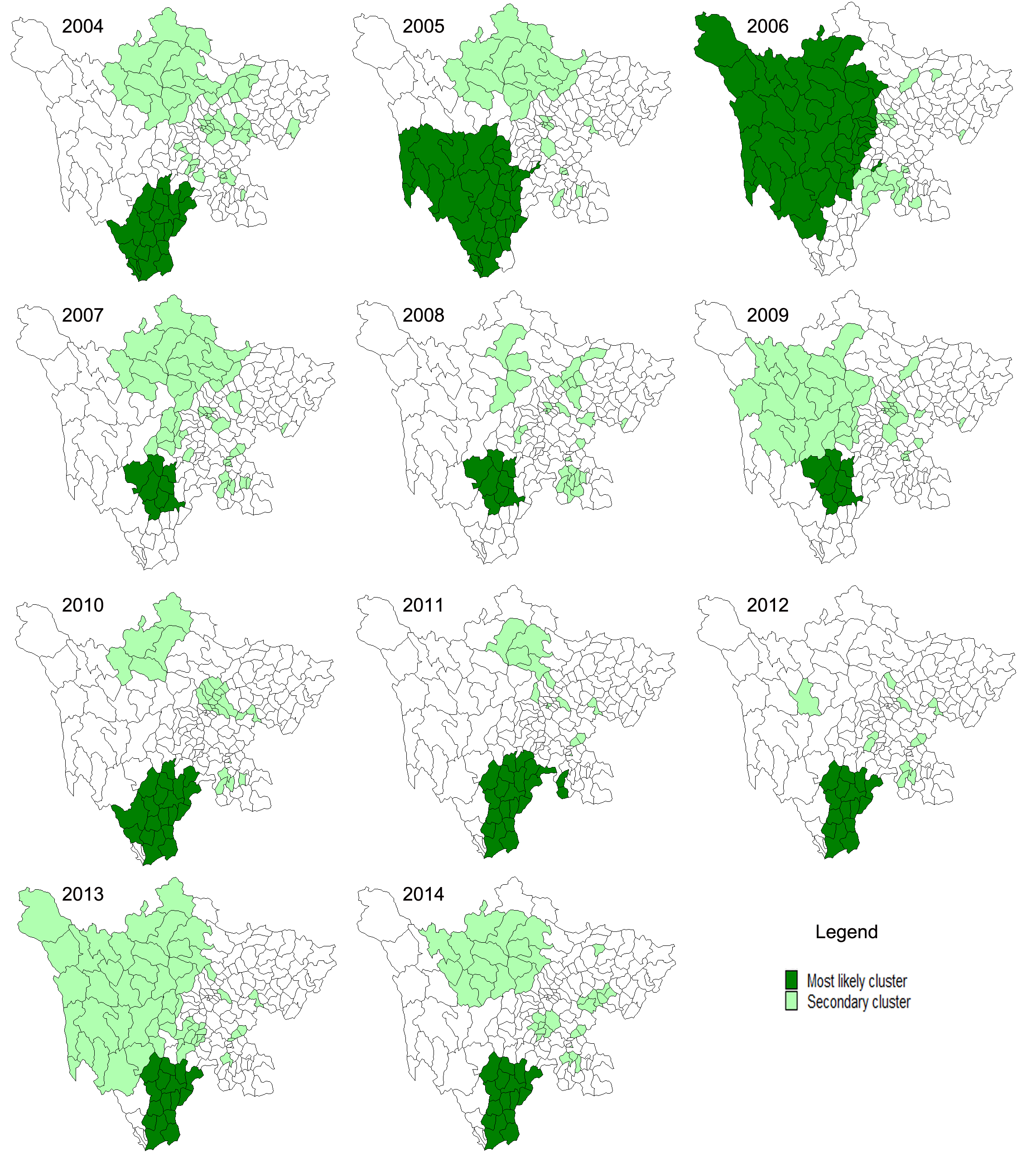


Supplementary Figure S1. Spatial-temporal clusters of BD in Sichuan, China, 2004-2014 (created with MapInfo Professional software version 7.0).


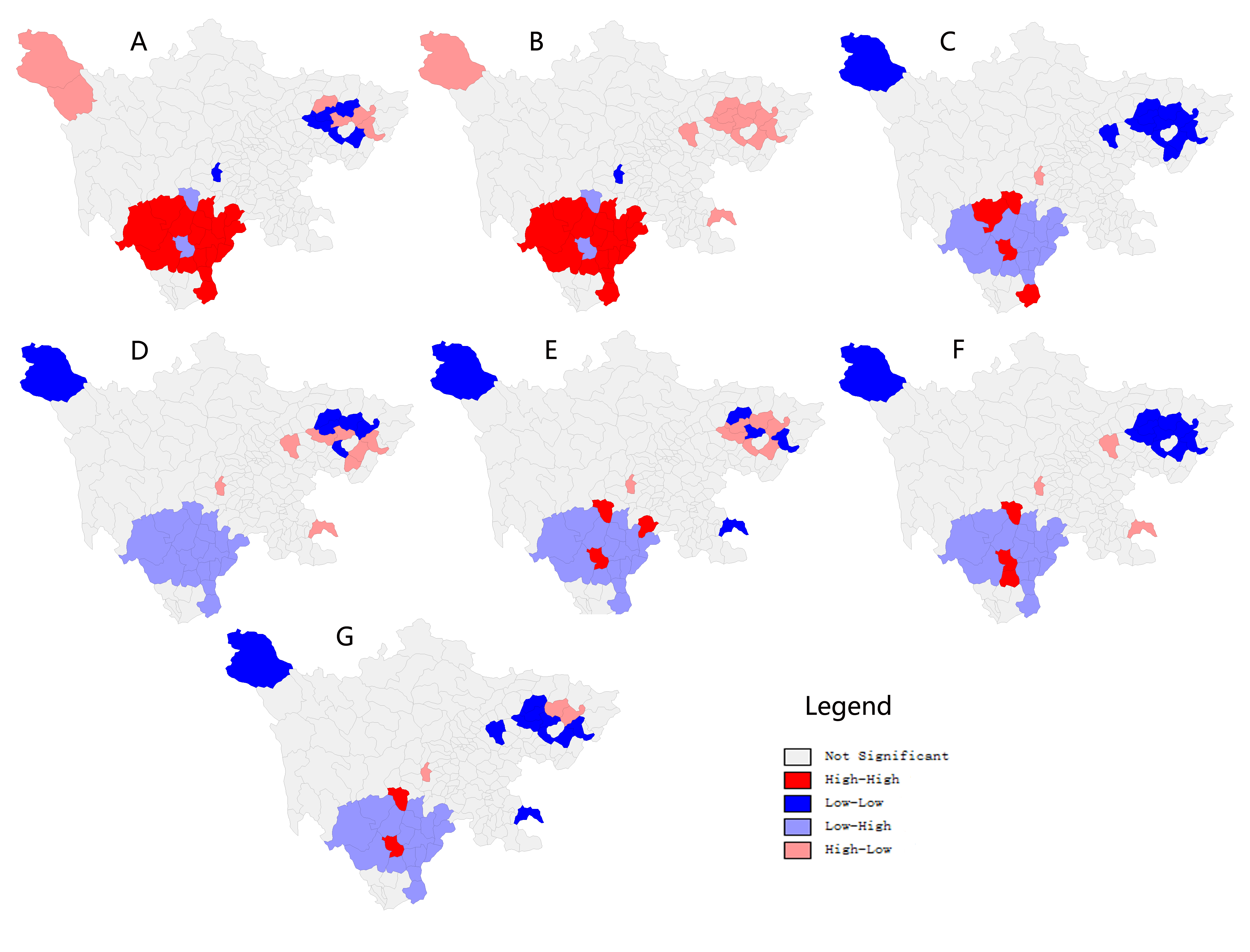


Supplementary Figure S2. Bivariate LISA cluster maps of socio-economic factors and BD incidence at county level in Sichuan province, 2012. A. Bivariate LISA cluster map of the proportion of primary industry and BD incidence. B. Bivariate LISA cluster map of the proportion of rural population and BD incidence. C. Bivariate LISA cluster map of Per capita GDP and BD incidence. D. Bivariate LISA cluster map of the proportion of secondary industry and BD incidence. E. Bivariate LISA cluster map of the proportion of tertiary industry and BD incidence. F. Bivariate LISA cluster map of number of beds in hospitals per thousand persons and BD incidence. G. Bivariate LISA cluster map of medical and technical personnel per thousand persons and BD incidence (created with GeoDa software version 1.3.28).
